# A Study on the Photothermal Catalytic Performance of Pt@MnO_2_ for O-Xylene Oxidation

**DOI:** 10.3390/molecules30214193

**Published:** 2025-10-27

**Authors:** Rong Qiao, Yanxuan Wang, Jiani Chen, Haotian Hu, Jiafeng Wei, Fukun Bi, Ye Zheng, Xiaodong Zhang

**Affiliations:** 1 School of Environment and Architecture, University of Shanghai for Science and Technology, Shanghai 200093, China; qr020607@163.com (R.Q.); wangyanxuan@163.com (Y.W.); chenhzyq@163.com (J.C.); huhaotian0109@126.com (H.H.); wjf6938@126.com (J.W.); bifukun@usst.edu.cn (F.B.); yezizheng4949@outlook.com (Y.Z.); 2 School of Health Science and Engineering, University of Shanghai for Science and Technology, Shanghai 200093, China; 3 Shanghai Non-Carbon Energy Conversion and Utilization Institute, Shanghai 200240, China

**Keywords:** photothermal catalytic, Pt, MnO_2_, o-xylene

## Abstract

Photothermal catalysis has emerged as a promising approach for the efficient and cost-effective removal of volatile organic compounds (VOCs). Pt@MnO_2_ catalysts have demonstrated excellent performance in the photothermal catalytic oxidation of VOCs. However, current research has predominantly focused on the interaction between Pt and MnO_2_, while often overlooking the influence of the MnO_2_ crystal phase. Therefore, in this study, we synthesized Pt supported on four crystal phases (α, β, γ, and δ) of MnO_2_ and established the structure–activity relationships through performance evaluation and characterization. Among the prepared catalysts, Pt@Mn[δ] exhibited excellent performance and possessed outstanding stability. Crystal structure characterization revealed that the larger specific surface area and lower crystallinity of Pt@Mn[δ] exposed more active sites. XPS analysis indicated the transformation of Mn^4+^ to Mn^3+^ on Pt@Mn[δ], leading to the formation of oxygen vacancies. O_2_-TPD and H_2_-TPR further confirmed the activation of lattice oxygen and the promoted redox cycle of Pt@Mn[δ]. UV-Vis DRS and electrochemical measurements demonstrated that Pt@Mn[δ] exhibited the most pronounced visible-light absorption, the highest photocurrent density, the lowest charge transfer resistance and superior charge carrier mobility. TD-GC-MS analysis indicated that o-xylene underwent alkylation and isomerization, with subsequent oxidation following the Mars–van Krevelen (MvK) mechanism.

## 1. Introduction

Volatile organic compounds (VOCs) are a major group air pollutants characterized by their complex composition and diverse emission sources, posing significant adverse effects to both human health and air quality [1,2]. Therefore, it is an urgent task to effectively remove VOCs. In recent years, the development of VOC removal technologies from industrial exhaust gas has attracted considerable attention. Current VOC elimination methods include adsorption, photocatalysis, plasma catalysis, thermal catalysis, and photothermal catalysis [3]. Among them, photothermal catalysis has emerged as a particularly promising approach, with higher efficiency and less energy consumption [4]. Catalysts serve as the core component of photothermal catalysis systems, and their performance directly governs the conversion efficiency of VOC eliminations from exhaust gas [5]. Therefore, substantial research endeavors have focused on developing highly efficient, cost-effective, and durable catalysts.

Over the years, various catalysts have been developed for the photothermal catalytic degradation of VOCs, mainly falling into two categories: transition metal oxides and noble metals such as Pd and Pt [6]. Despite the high efficacy of noble metals in VOC oxidation, their practical application is limited by high cost and scarcity [7]. In contrast, transition metal oxides offer advantages such as low cost, poison resistance, and sintering stability, making them attractive alternatives. However, they generally suffer from lower activity, poorer stability, and higher susceptibility to deactivation in complex atmospheres, restricting their widespread use [8]. To address these limitations, supported noble metal catalysts—where noble metals are dispersed on various supports—have gained increasing attention. The dispersion of noble metal nanoparticles maximizes active sites and enhances synergistic effects with the support, significantly boosting catalytic performance [9]. For example, Li et al. [10] prepared a Pt/[TiN@TiO_2_] core–shell catalyst that exhibited broadened light absorption and high photothermal efficiency, achieving complete toluene degradation under 500 mW·cm^−2^ light irradiation. Similarly, Fan et al. [11] developed a Pd/Fe-TiO_2_ catalyst with dual-active sites, where the incorporation of Pd and Fe promoted charge separation and reactive oxygen species generation, leading to significantly improved toluene degradation.

Supported Pt catalysts are regarded as highly promising for VOC oxidation due to their high activity and stability. In such systems, the Pt–support interface facilitates the formation of Pt^0^/Pt^δ+^ active species, which play a key role in enhancing oxidation reactivity and modulating catalyst properties [9]. For instance, Wang et al. [12] developed Pt/N–TiO_2_–H_2_, which contained a high proportion of Pt^0^ and achieved 98.4% toluene conversion under 260 mW·cm^−2^ light irradiation. The choice of support is crucial in photothermal catalysis. MnO_2_ has attracted wide interest owing to its broad light absorption from visible to NIR regions and multivalent manganese species, enabling both photocatalytic and thermal catalytic functions [13,14,15]. Yu et al. [16] prepared 1Pt/MO by depositing Pt nanoparticles on MnO_2_, attaining complete toluene degradation under 200 mW·cm^−2^ light. Wang et al. [17] further constructed a Pt single-atom catalyst on δ-MnO_2_, where strong Pt–support electronic interaction activated lattice oxygen and promoted intermediate conversion. Despite these advances, most previous studies have emphasized noble metal–support interactions, while the influence of the support’s intrinsic structure on catalytic performance has often been overlooked. MnO_2_ consists of [MnO_6_] octahedra as fundamental building units, can assemble into diverse structural architectures through different connectivity patterns, thereby exhibiting multiple crystal phases [18]. Distinct polymorphs of MnO_2_ possess markedly different physicochemical properties, suggesting that the structure–activity relationship can substantially modulate catalytic behavior.

Herein, MnO_2_[α, β, γ, and δ] with different crystalline phases were synthesized via a hydrothermal method. Subsequently, Pt was immobilized onto MnO_2_ supports using the NaBH_4_ reduction method, resulting in the formation of Pt@Mn[α], Pt@Mn[β], Pt@Mn[γ], and Pt@Mn[δ] catalysts. A series of characterization techniques were utilized to probe the structural characteristics of the synthesized catalysts. Subsequently, we evaluated the photothermal catalytic activity of Pt@Mn[α, β, γ, and δ] in the oxidation of o-xylene. The degradation pathway of o-xylene was further elucidated using the thermal desorption–gas chromatography–mass spectrometry (TD-GC-MS).

## 2. Results and Discussion

### 2.1. Structure Characterization

**Figure 1:**
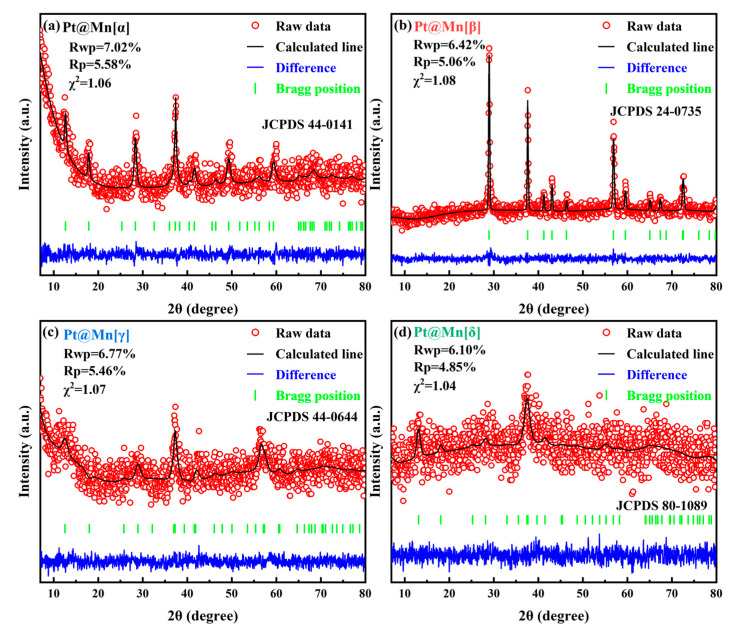
Rietveld refined XRD patterns for (**a**) Pt@Mn[α], (**b**) Pt@Mn[β], (**c**) Pt@Mn[γ] and (**d**) Pt@Mn[δ].

The crystallographic structures of Pt@Mn[α, β, γ, and δ] catalysts with different polymorphs were characterized by XRD, and Rietveld refinement was performed on the obtained patterns. Figure 1 shows that prepared catalysts displayed diffraction patterns attributable only to their specific MnO_2_ phases [19,20,21,22]. Moerover, the XRD peaks of Pt@Mn[α] were shown at 49.7°, 41.8°, 37.6°, 28.5°, 17.9°, and 12.2°, which can be corresponded to the (411), (301), (211), (310), (200), and (110) planes. The diffraction peaks observed at 66.2°, 37.4°, 25.6°, and 13.1° can be assigned to the (020), (−111), (002), and (001) crystal planes of Pt@Mn[δ]. For Pt@Mn[γ], the peaks observed at 45.6°, 37.2°, and 22.7° correspond to the (300), (131), and (120) planes. Meanwhile, the peaks at 43.1°, 37.6°, and 29.0° were assigned to the (111), (101), and (110) planes of Pt@Mn[β]. This phase purity confirmed that no significant crystal transformation occurred during Pt deposition. In addition, the absence of detectable PtO_x_ diffraction peaks suggested a high dispersion of Pt species on the catalyst surfaces. Notably, Pt@Mn[β] displayed substantially sharper and more intense diffraction peaks compared to Pt@Mn[α], Pt@Mn[γ] and Pt@Mn[δ]. In contrast, the comparatively broad peaks observed for Pt@Mn[δ] indicated poor crystallinity, primarily attributed to synthesis-induced structural defects that generate pronounced disorder along specific crystallographic directions [23]. Then the grain size was calculated from the Full Width at Half Maxima (HWFM) of the major crystallographic plane, as shown in Table 1. The calculated grain sizes followed the order: Pt@Mn[β] (25.6) > Pt@Mn[α] (18.9) > Pt@Mn[γ] (15.1) > Pt@Mn[δ] (10.3). Consequently, Pt@Mn[δ] possessed the smallest grain size, which might due to the formation of surface defect sites [24].

**Figure 2:**
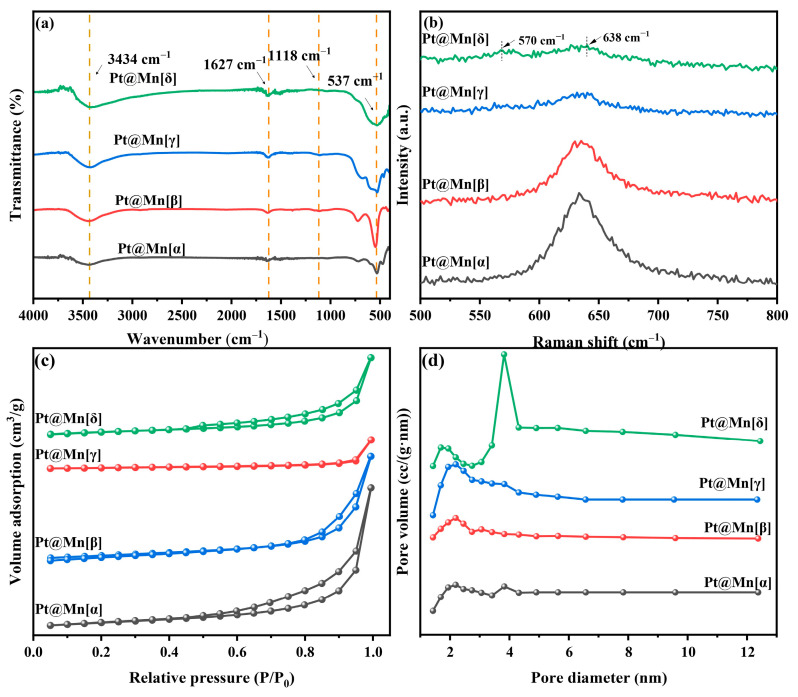
(**a**) FT-IR spectra, (**b**) Raman patterns, (**c**) N_2_ adsorption and desorption isotherms and (**d**) pore size distribution curves for Pt@Mn[α, β, γ, and δ].

The functional groups on the prepared catalysts were identified by FT-IR spectroscopy, Figure 2a. The observed bands at approximately 3434 cm^−1^ and 1627 cm^−1^ are characteristic of O–H stretching and bending vibrations, respectively, indicating the presence of adsorbed water [25]. Furthermore, bands were observed at approximately 1118 cm^−1^ and 543 cm^−1^, which were assigned to Mn–O–H coordination and the stretching vibration of Mn–O/Mn–O–Mn, respectively [26]. Among them, the Mn-O bond vibration at 543 cm^−1^ exhibited the highest intensity for Pt@Mn[β], while it was less intense for Pt@Mn[γ] and Pt@Mn[δ].

Raman spectroscopy was employed to elucidate the crystal structures of the prepared catalysts. The observed peaks in the 500–700 cm^−1^ range were consistent with [MnO_6_] octahedra vibrations. For Pt@Mn[δ], the spectrum consisted of two primary bands at approximately 570 and 638 cm^−1^, the latter of which was ascribed to the symmetric stretching vibration of the Mn–O bond [27]. The Raman band at 570 cm^−1^ was attributed to the in-plane Mn–O stretching vibration of the [MnO_6_] sheets. Notably, the broad band around 638 cm^−1^ observed for the prepared catalysts demonstrated that the incorporation of Pt did not change the MnO_2_ structure [28]. The Raman spectrum of prepared catalysts in Figure 2b exhibited a characteristic band near 638 cm^−1^, which is attributed to the Mn-O vibration within the [MnO_6_] octahedral framework. Furthermore, the different crystal phases exhibit distinctive vibrational modes (Pt@Mn[α]: breathing vibrations; Pt@Mn[β]: symmetric stretching vibrations; Pt@Mn[γ]: symmetric stretching vibrations; Pt@Mn[δ]: symmetric stretching vibration). Notably, the Pt@Mn[δ] catalyst exhibited the lowest band intensity among the series, suggesting a lower degree of crystallinity. This observation is consistent with the broader XRD patterns observed for this material (Figure 1), further supporting its relatively disordered structure. Lower crystallinity generally facilitates the exposure of a greater number of active sites, thereby enhancing the catalytic activity [29].

The N_2_ adsorption–desorption isotherms of Pt@Mn[α, β, γ, and δ] catalysts are presented in Figure 2c. Pt@Mn[α], Pt@Mn[γ], and Pt@Mn[δ] displayed typical Type IV adsorption–desorption isotherms accompanied by H3-type hysteresis loops, while Pt/β-MnO_2_ aligned well with a Type III isotherm, also featuring the H3 hysteresis loop. These results collectively indicated the presence of mesoporous structures in all prepared catalysts [30]. The textural properties of the catalysts, including specific surface area, pore size distribution, and average pore size, are listed in Table 1. The BET surface areas varied significantly, ranging from 13.2 m^2^·g^−1^ for Pt@Mn[α] to 59.7 m^2^·g^−1^ for Pt@Mn[δ]. The adsorption capacity of o-xylene was strongly influenced by the specific surface area, which governed the population of accessible active sites [13]. A correlation was observed between the pore volume and the specific surface area. Figure 2d displayed the corresponding pore size distribution calculated by the Barrett–Joyner–Halenda (BJH) method. Conversely, Pt@Mn[δ] and Pt@Mn[γ] exhibited broader pore size distributions with larger average pore diameters, suggesting more heterogeneous pore structures.

### 2.2. Surface Chemical Properties Analysis

**Figure 3:**
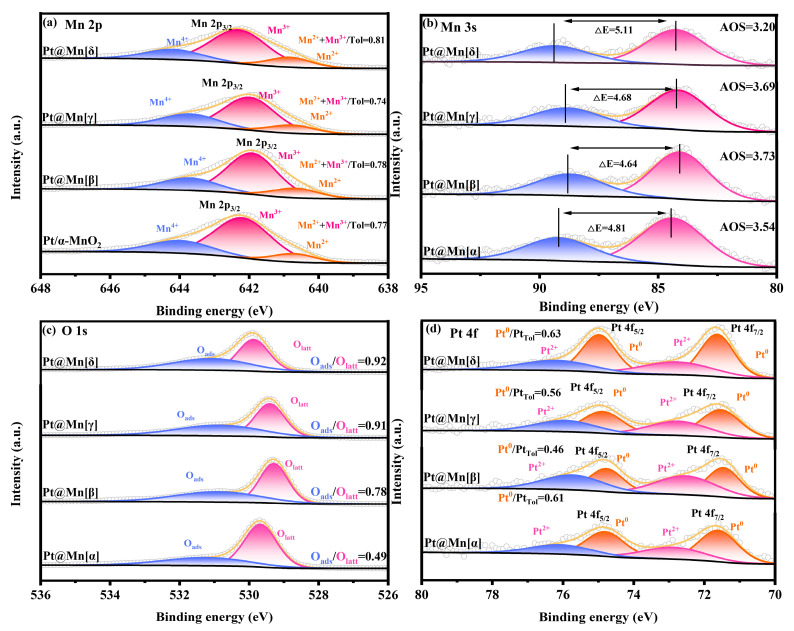
XPS spectra of Pt@Mn[α, β, γ, and δ]: (**a**) Mn 2p, (**b**) Mn 3s, (**c**) O 1s, and (**d**) Pt 4f.

The Mn 2p XPS spectra of the Pt@Mn[α, β, γ, and δ] catalysts are presented in Figure 3a. Spectral deconvolution revealed three components at binding energies of 642.7 eV, 641.7 eV, and 640.4 eV, corresponding to Mn^4+^, Mn^3+^, and Mn^2+^ species, respectively [31]. The concentrations of low-valent Mn (Mn^2+^ and Mn^3+^) in the different catalysts were summarized in Table 1. From Table 1, it can be seen that Pt@Mn[δ] possessed the highest relative concentration of low-valence Mn (Mn^2+^ and Mn^3+^), which was 0.81. According to previous studies, the low-valent Mn^2+^ species and Mn^3+^ species represent the presence of surface oxygen vacancies in the catalyst, which is due to the need to balance the charge during the process of Mn^4+^ changing from a high-valent state to a low-valent state [32]. The Mn 3s XPS spectra (Figure 3b) were used to quantify the average oxidation state (AOS) of Mn, providing further evidence for the existence of low-valent Mn species. The calculated average oxidation states (AOS) of the synthesized samples are presented in Table 1. Notably, Pt@Mn[δ] exhibits the lowest AOS with a value of 3.20. This finding aligns with the established mechanistic understanding that the reduction of Mn^4+^ to lower oxidation states necessitates the generation of oxygen vacancies to maintain charge neutrality. The intrinsic crystal defects and tunnel hybridization in Pt@Mn[δ] were favorable for stabilizing more Mn^2+^/^3+^ on the surface, consequently enhancing the formation of oxygen vacancies. The XPS spectra of O 1s of Pt@Mn[α, β, γ, and δ] samples are shown in Figure 3c. As displayed in Figure 3c, the O 1s XPS spectra of the Pt@Mn[α, β, γ, and δ] samples exhibited two peaks at 529.4 eV and 531.1 eV, which were attributed to lattice oxygen (O_latt_) and surface-adsorbed oxygen (O_ads_), respectively [33]. The ratios of O_ads_/O_latt_ for four Pt@Mn[α, β, γ, and δ] samples were calculated based on the peak areas. Typically, oxygen vacancies serve as active sites on the material surface and exhibit a strong tendency to form O_ads_, leading to the formation of adsorbed oxygen species. Consequently, higher ratio of O_ads_/O_latt_ implies greater concentration of oxygen vacancies [34]. As summarized in Table 1, Pt@Mn[δ] showed the greatest O_ads_/O_latt_ proportion (0.92), which indicated a higher concentration of oxygen vacancies in Pt@Mn[δ]. Oxygen vacancies facilitated the activation of gaseous oxygen, promoted the regeneration of lattice oxygen, and accelerated the redox cycle in the Mars–van Krevelen (MvK) mechanism, thereby enhancing the overall catalytic activity [35]. The XPS spectra of Pt 4f orbitals for Pt@Mn[α, β, γ, and δ] catalysts were presented in Figure 3d, revealing that Pt species primarily exist in metallic Pt^0^ and oxidized Pt^2+^ states. The Pt^0^/Pt_total_ ratios were presented in Table 1. Quantitative analysis of the catalyst series revealed that the Pt@Mn[δ] catalyst possessed the highest proportion of metallic Pt^0^, with a Pt^0^/Pt_total_ of 0.63. This finding was consistent with previous reports establishing a positive correlation between Pt^0^ content and catalytic activity [36].

**Figure 4:**
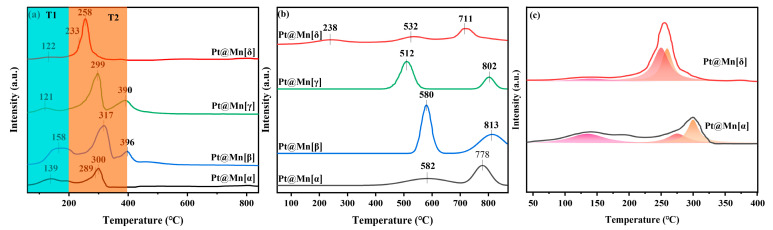
(**a**) H_2_-TPR, (**b**) O_2_-TPD profile of Pt@Mn[α, β, γ, and δ], and (**c**) deconvolution analysis on the overlapping H_2_-TPR peaks of Pt@Mn[α] and Pt@Mn[δ].

The reducibility of catalysts is crucial to the catalytic performance. Therefore, H_2_-TPR was employed to characterize the catalysts and is presented in Figure 4a. All catalysts exhibited a similar profile of reduction peaks, which were categorized into a low-temperature peak (T_1_, 0–200 °C) and a medium-temperature peak (T_2_, 200–400 °C). The peak in the T_1_ region was primarily due to the reduction of PtO_x_, while the peak in the T_2_ region was primarily due to the stepwise reduction in surface Mn^4+^ in MnO_2_. Specifically, the peak in the T_1_ region arose from the reduction of PtO_x_ to metallic platinum (Pt^0^), and the broad peak in the T_2_ region corresponded to the multi-step reduction process of Mn^4+^ → Mn^3+^ → Mn^2+^ [37,38]. A comparison of the peak in the T_1_ region among the prepared catalysts revealed that Pt@Mn[δ] possessed the smallest peak area, indicating the lowest content of PtO_x_ and the highest proportion of metallic Pt^0^. This conclusion was consistent with the results obtained from XPS analysis. During the medium-temperature stage, the peak in the T_1_ region of the prepared catalysts exhibited pronounced differences. For Pt@Mn[δ] and Pt@Mn[α], the overlapping reduction peaks suggested a rapid reduction process of Mn^4+^ (Figure 4). In contrast, Pt@Mn[β] and Pt@Mn[γ] displayed distinct, separated peaks, clearly indicating a stepwise reduction pathway of Mn^4+^ proceeding at a comparatively slower rate. Moreover, the peak in the T_2_ region for Pt@Mn[δ] appeared at a lower temperature compared to those of Pt@Mn[γ], Pt@Mn[β], and Pt@Mn[α], which could be explained by the enhanced hydrogen spillover effect from Pt^0^ to the MnO_x_ support [9]. Specifically, the high concentration of metallic Pt^0^ on Pt@Mn[δ] facilitated the dissociation of H_2_ into active hydrogen species (H·), which acted as potent reducing agents to reduce Mn^4+^ via the metal–support interface. These results demonstrated that Pt@Mn[δ] demonstrated the greatest reducing capability among all synthesized samples.

The oxidative activity of catalysts is directly related to the amount and reactivity of the active oxygen species. Hence, the Pt@Mn[α, β, γ, and δ] samples were characterized by O_2_-TPD and are shown in Figure 4b. For the catalytic oxidation of VOCs, researchers have focused on the peaks below 600 °C [16]. Therefore, the oxygen species with desorption temperatures below 600 °C are the primary contributors to the catalytic process [39,40]. Specifically, the peak below 300 °C was attributed to the desorption of oxygen molecule anions (O_2_^−^) adsorbed on oxygen vacancies, while the peak between 300 and 600 °C was attributed to the desorption of oxygen anions (O^−^) in the lattice oxygen. Observing the desorption peaks, it was found that Pt@Mn[δ] was significantly different from the other three catalysts. This indicated that there were differences in the oxygen species characteristics among different crystal forms of MnO_2_. Specifically, Pt@Mn[δ] had a distinct broad peak at 238 °C, indicating that the oxygen species adsorbed on oxygen vacancies on the Pt@Mn[δ] surface participate in the catalytic process at a lower temperature [6]. However, no desorption peaks were observed in this temperature range for Pt@Mn[α], Pt@Mn[β], and Pt@Mn[γ] catalysts, suggesting their poorer oxidation ability. Additionally, desorption peaks were observed for all prepared catalysts between 300 and 600 °C, indicating that lattice oxygen also participated in the reaction.

### 2.3. Detection of Optical Properties

**Figure 5:**
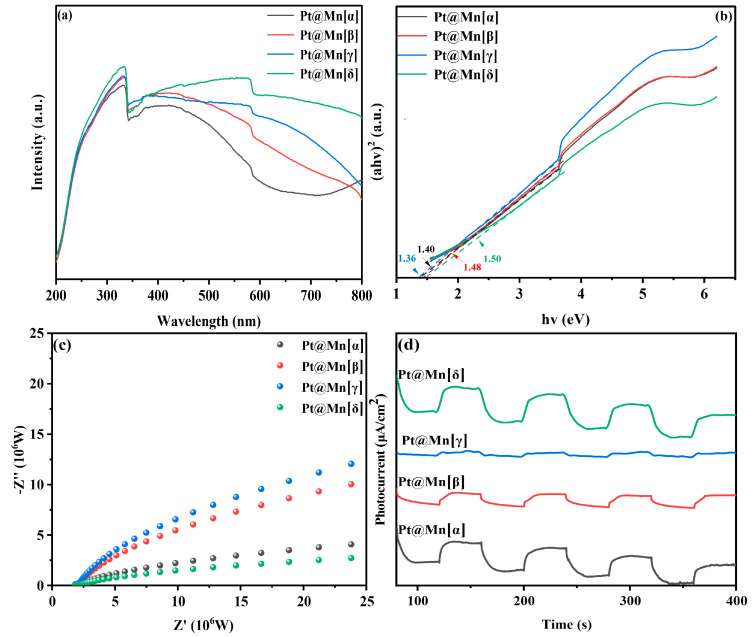
(**a**) UV-vis DRS spectra; (**b**) Tauc plot; (**c**) EIS plots; and (**d**) photocurrents of Pt@Mn[α, β, γ, and δ].

The light absorption characteristics of catalytic materials play a crucial role in determining their photothermal catalytic performance. The optical properties of the Pt@Mn[α, β, γ, and δ] catalysts were studied using UV-vis diffuse reflectance spectroscopy (DRS). As shown in Figure 5a, all catalysts exhibited strong broadband absorption across the entire measured spectral range, which can be attributed to their intrinsic black coloration. Notably, the Pt@Mn[δ] exhibited significantly enhanced light absorption capabilities across both ultraviolet and visible spectral regions compared to other catalysts. The optical band gaps (Eg) of the catalysts were determined using Tauc plot analysis of the absorbance data (Figure 5b). The calculated Eg values followed the order Pt@Mn[δ] (1.50 eV) > Pt@Mn[β] (1.48 eV) > Pt@Mn[α] (1.40 eV) > Pt@Mn[γ] (1.36 eV). To evaluate the charge carrier dynamics, we conducted photocurrent and electrochemical impedance (EIS) measurements The photocurrent response (Figure 5c) revealed the following trend in photoinduced electron density: Pt@Mn[γ] > Pt@Mn[β] > Pt@Mn[α] > Pt@Mn[δ]. Notably, Pt@Mn[δ] exhibited a significantly enhanced photocurrent response compared to other catalysts, suggesting that Pt incorporation effectively suppressed the recombination of photogenerated electron–hole pairs [41,42]. The EIS results (Figure 5d) demonstrated good correlation with the photocurrent measurements. The Nyquist plots showed that Pt@Mn[δ] possessed the smallest arc radius, indicating the lowest charge transfer resistance among the tested catalysts and superior charge carrier mobility with minimized recombination losses [43,44]. These characteristics collectively contribute to the enhanced photocatalytic performance observed for Pt@Mn[δ].

### 2.4. Catalytic Activity

**Figure 6:**
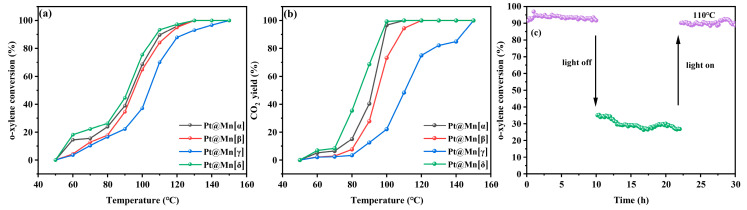
(**a**) o-xylene conversion and (**b**) CO_2_ yield of Pt@Mn[α, β, γ, and δ] and (**c**) stability of Pt@Mn[δ].

As shown in Figure 6a, the o-xylene oxidation conversion over the Pt@Mn[α, β, γ, and δ] catalysts remained below 30% at temperatures under 80 °C. The conversion of o-xylene increased rapidly once the reaction temperature exceeded 80 °C. Complete o-xylene degradation was achieved over Pt@Mn[α] at 147 °C, Pt@Mn[β] at 149 °C, Pt@Mn[γ] at 150 °C, and Pt@Mn[δ] at 145 °C. The catalytic activity followed the order: Pt@Mn[δ] > Pt@Mn[α] > Pt@Mn[β] > Pt@Mn[γ], with Pt@Mn[δ] exhibiting the highest performance. For quantitative comparison, Table 2 summarized the characteristic temperatures required to achieve 10%, 50%, and 90% o-xylene conversion (T_10_, T_50_, and T_90_). T_10_, T_50_, and T_90_ values exhibited the same trend—Pt@Mn[δ] < Pt@Mn[α] < Pt@Mn[β] < Pt@Mn[γ] —confirming the superior low-temperature activity of Pt@Mn[δ]. These results clearly demonstrate that Pt@Mn[δ] possesses exceptional catalytic performance for the complete oxidation of o-xylene. In addition, the CO_2_ yield at equivalent reaction temperature was evaluated, as presented in Figure 6b. At 80 °C, the Pt@Mn[δ], Pt@Mn[α], Pt@Mn[β], and Pt@Mn[γ] catalysts yielded CO_2_ at rates of 39%, 27%, 8%, and 4%, respectively. Although the CO_2_ yield progressively increased with rising temperature, the activity sequence remained unchanged throughout the tested range. To further investigate the stability of the Pt@Mn[δ] catalyst, photothermal tests were conducted at 110 °C under simulated sunlight illumination. As presented in Figure 6c, the o-xylene conversion exhibited excellent stability during the initial 10 h illumination period, decreasing only slightly from 93% to 90%. However, when the light source was removed, the conversion dropped significantly to approximately 20%. After maintaining this dark condition for 10 h, the light was reintroduced, and the o-xylene conversion promptly recovered to 90% with subsequent stable performance. These results clearly demonstrated that the Pt@Mn[δ] catalyst maintains exceptional stability under photothermal catalytic conditions, with its activity being light-dependent and fully reversible.

### 2.5. Intermediates and Reaction Mechanism

**Figure 7:**
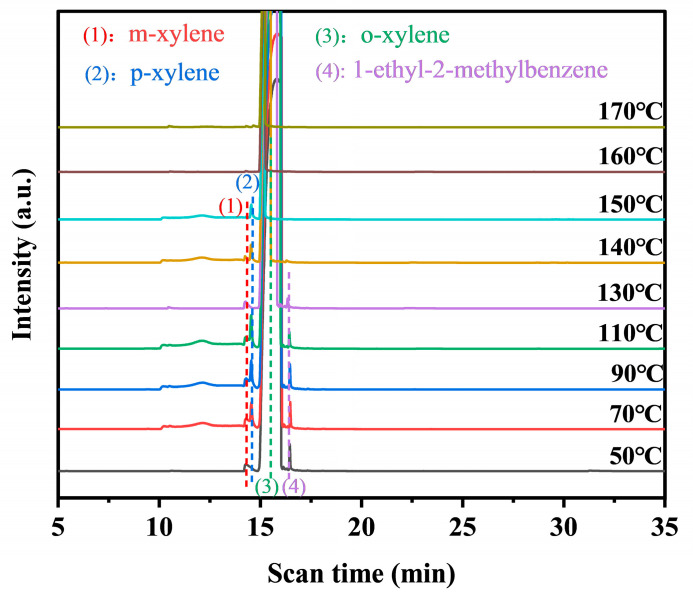
TD-GC-MS of Pt@Mn[δ] under photothermal catalysis.

**Figure 8:**
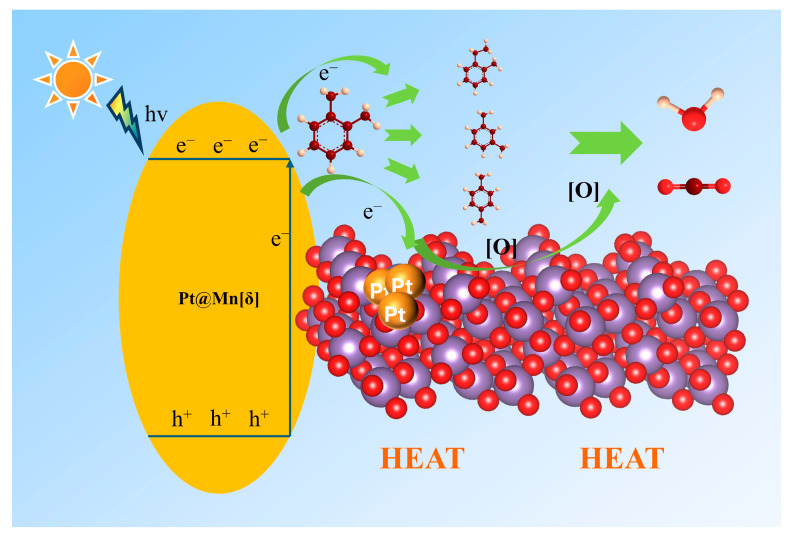
Schematic reaction mechanism under photothermal catalysis.

The intermediates formed during the photothermal catalysis oxidation of o-xylene on Pt@Mn[δ] were identified by TD-GC-MS (Figure 7). Three probable intermediates were detected: m-xylene, p-xylene, and 1-ethyl-2-methylbenzene. The formation of m- and p-xylene was attributed to acid-catalyzed isomerization (Friedel––Crafts reaction), proceeding via intramolecular or intermolecular methyl shifts, followed by protonation–deprotonation prior to desorption. In addition, 1-ethyl-2-toluene was produced by the alkylation of o-xylene [45]. Based on the experimental findings, a potential photothermal synergistic mechanism was illustrated in Figure 8. The process initiated with oxygen activation, wherein O_2_ molecules acquired electrons from the catalyst surface to form active oxygen species. Subsequently, MnO_2_ compensated for electron loss by extracting electrons from adsorbed o-xylene or reaction intermediates. As electron transfer kinetics were thermally enhanced, elevated temperatures facilitated more efficient activation. Additionally, increased temperature improved surface oxygen mobility, promoting the transfer of proton-activated oxygen species, thereby accelerating the overall reaction process. Consequently, the probable degradation pathway of o-xylene proceeded primarily through intermediates such as m-xylene, p-xylene, and 1-ethyl-2-methylbenzene, ultimately leading to complete mineralization into CO_2_ and H_2_O.

## 3. Experimental Section

### 3.1. Chemicals and Reagents

KMnO_4_ (A.R.), MnSO_4_·H_2_O (A.R.) and H_2_PtCl_6_·6H_2_O were purchased from Sinopharm Chemical Reagent Co., Ltd. (Shanghai, China). (NH_4_)_2_S_2_O_8_ (A.R.) was purchased from Macklin Biochemical Co., Ltd. (Shanghai, China). All chemicals were of analytical grade and used without further purification.

### 3.2. Catalysts Preparation

α-, β-, γ-, and δ-MnO_2_ were synthesized by the hydrothermal method. The specific polymorph of MnO_2_ was determined primarily by the selection of the precursor and tailored through precise control of the hydrothermal temperature. Pt was immobilized onto MnO_2_ supports using the NaBH_4_ reduction method, resulting in the formation of Pt@Mn[α], Pt@Mn[β], Pt@Mn[γ], and Pt@Mn[δ] catalysts. The comprehensive experimental details are provided in the Supporting Information.

### 3.3. Structural Characterization

The prepared catalysts were characterized through multiple experiments such as X-ray diffraction (XRD, Billerica, MA, USA), Fourier transform infrared (FT-IR, Waltham, MA, USA), Raman spectroscopy (Kyoto, Japan), N_2_ adsorption–desorption experiment (Boynton Beach, FL, USA), X-ray photoelectron spectroscopy (XPS, Waltham, MA, USA), the solid-state UV-Vis spectroscopy, electrochemical measurements, hydrogen programmed-temperature reduction (H_2_-TPR, Boynton Beach, FL, USA) test, the oxygen programmed-temperature desorption (O_2_-TPD, Boynton Beach, FL, USA) test and thermal desorption–gas chromatograph–mass spectrometer (TD-GC-MS, Agilent Technologies, Santa Clara, CA, USA). Comprehensive experimental details can be found in the Supporting Information.

### 3.4. Catalytic Reaction

The catalytic performance was assessed in a quartz microreactor. The reaction system contained o-xylene at 300 ppm and oxygen at 21%, along with an optical density of 297.3 mW·cm^−2^. The detailed process could be found in the Supporting Information.

## 4. Conclusions

In this work, four Pt@Mn[α, β, γ, and δ] catalysts with distinct crystal phases were synthesized via hydrothermal treatment followed by NaBH_4_ reduction. Multiple characterization techniques confirmed that Pt@Mn[δ] had the largest specific surface area, the smallest grain size, the highest oxygen vacancies, and superior low-temperature redox properties. Furthermore, the Pt@Mn[δ] catalyst exhibited the most efficient photogenerated carrier separation, the strongest photoresponse capability, and the broadest light absorption range among all preparedd samples. These advantages resulted in the excellent photothermal performance of Pt@Mn[δ] in o-xylene oxidation. The probable degradation of o-xylene proceeded through a pathway that primarily involved the following steps: o-xylene → 1-ethyl-2-methylbenzene, p-xylene and m-xylene → CO_2_ and H_2_O.

## Figures and Tables

**Table 1:** Chemical and physical properties of Pt@Mn[α, β, γ, and δ].

Samples	Average Grain Size (nm) ^a^	S_BET_ (m^2^/g) ^b^	V_total_ (cc/g) ^c^	D (nm) ^d^	Pt^0^/Pt_total_ ^e^	(Mn^2+^ + Mn^3+^)/Mn_total_ ^f^	O_ads_/O_latt_ ^g^	AOS ^h^
Pt@Mn[α]	18.9	50.3	0.29	1.4–4.9	0.61	0.77	0.49	3.54
Pt@Mn[β]	25.6	13.9	0.06	1.7–5.6	0.46	0.78	0.78	3.73
Pt@Mn[γ]	15.1	54.1	0.31	1.4–4.9	0.56	0.74	0.91	3.69
Pt@Mn[δ]	10.3	59.7	0.23	1.2–4.9	0.63	0.81	0.92	3.20

^a^ Grain size was calculated from the HWFM of the major crystallographic plane surface. ^b^ BET specific surface. ^c^ Total pore volume measured at P/P_0_ = 0.99; ^d^ Pore size distribution was calculated by the BJH method. ^e^ Pt^0^/Pt_total_ was derived from integrated peak area in deconvoluted XPS spectra. ^f^ (Mn^2+^ + Mn^3+^)/Mn_total_ was derived from integrated peak area in deconvoluted XPS spectra. ^g^ O_ads_/O_latt_ was derived from integrated peak area in deconvoluted XPS spectra. ^h^ AOS was calculated based on the XPS spectra of Mn 3s, AOS = 8.956 − 1.126 ΔEs (eV).

**Table 2:** Catalytic performance of Pt@Mn[α, β, γ, and δ].

Samples	T_10_ (°C)	T_50_ (°C)	T_90_ (°C)
Pt@Mn[α]	57	95	110
Pt@Mn[β]	70	100	118
Pt@Mn[γ]	73	110	130
Pt@Mn[δ]	55	94	109

## Data Availability

The authors declare that all data supporting the findings of this study are available within the article.

## Supplementary Materials

The following supporting information can be downloaded at: https://www.mdpi.com/article/10.3390/molecules30214193/s1, Ref. [46] is cited in Supplementary Materials.
