# Supplementary material for: A Study on the Photothermal Catalytic Performance of Pt@MnO2 for O-Xylene Oxidation
Source: Molecules. 2025 Oct 27;30(21):4193. doi: 10.3390/molecules30214193 (PMC12609410; doi:10.3390/molecules30214193)
Supplement: Supplementary file 1 [file molecules-30-04193-s001.zip › molecules-3929334-supplementary.pdf]

Supporting Information

# A Study on the Photothermal Catalytic Performance of Pt@MnO$_2$ for O-Xylene Oxidation

**Rong Qiao [1], Yanxuan Wang [1], Jiani Chen [1], Haotian Hu [1], Jiafeng Wei [1], Fukun Bi [1,2], Ye Zheng [1] and Xiaodong Zhang [1,3,*]**

[1] School of Environment and Architecture, University of Shanghai for Science and Technology,
Shanghai 200093, China; qr020607@163.com (R.Q.); wangyanxuan@163.com (Y.W.); chenhzyq@163.com (J.C.); huhaotian0109@126.com (H.H.); wjf6938@126.com(J.W.); bifukun@usst.edu.cn (F.B.)yezizheng4949@outlook.com (Y.Z.)
[2] School of Health Science and Engineering, University of Shanghai for Science and Technology,
Shanghai 200093, China
[3] Shanghai Non-carbon Energy Conversion and Utilization Institute, Shanghai 200240, China
* Correspondence: fatzhxd@126.com; Tel.: +86-159-2126-7160

(1) Synthesis of α-, β-, γ-, and δ-MnO$_2$

(2) **S**ynthesis of Pt supported on MnO$_2$ catalysts

(3) Structural characterization

(4) Catalytic reaction

*(1) Synthesis of α-, β-, γ-, and δ-MnO$_2$*

MnO$_2$[α, β, γ and δ] were synthesized hydrothermally according to the literature [1,2]. For α-MnO$_2$, a homogeneous mixture was prepared by stirring 1.25 g KMnO$_4$ and 0.53 g MnSO$_4$·H$_2$O in 80 mL deionized water for 30 min. This mixture was immediately transferred to a 100 mL Teflon-lined autoclave, sealed, and heated at 160 °C for 12 h. After cooling naturally to room temperature, the precipitate was collected by centrifugation, washed repeatedly with deionized water, and dried at 80 °C for 4 h. Subsequent calcination at 360 °C for 2 h yielded phase-pure α-MnO$_2$ as a brown crystalline powder.

For β-MnO2, a solution containing 1.69 g MnSO4·H2O and 2.28 g (NH4)2S2O8 in 80 mL deionized water was stirred for 30 min. Following the same transfer, sealing, and hydrothermal procedure (140 °C for 12 h), the cooled product underwent identical centrifugation, washing, drying (80 °C for 4 h), and calcination (360 °C for 2 h) steps, resulting in β-MnO2 as a black crystalline powder.

γ-MnO2 was synthesized by stirring 3.38 g MnSO4·H2O and 4.58 g (NH4)2S2O8 in 80 mL deionized water for 30 min. The mixture was hydrothermally treated at 90 °C for 24 h in an autoclave. After

cooling, the product was processed identically (centrifugation, washing, drying at 80 °C for 4 h, calcination at 360 °C for 2 h) to yield highly crystalline black γ-$MnO_2$ powder.

δ-$MnO_2$ was obtained using a mixture of 1.5 g $KMnO_4$ and 0.28 g $MnSO_4 \cdot H_2O$ in 80 mL deionized water, stirred for 30 min. Hydrothermal treatment was performed at 160 °C for 12 h. The subsequent isolation, washing, drying (80 °C for 4 h), and calcination (360 °C for 2 h) steps yielded phase-pure δ-$MnO_2$ as a black crystalline powder.

*(2) Synthesis of Pt supported on MnO2 catalysts*

An aqueous $H_2PtCl_6 \cdot 6H_2O$ solution was prepared by dissolving 625.92 μL $H_2PtCl_6 \cdot 6H_2O$ (20 mg/mL) in 20 mL deionized water (loading of approximately 0.5 wt%) under vigorous magnetic stirring for 15 min. Subsequently, 0.5 g of each synthesized $MnO_2$[α, β, γ and δ] were individually added to this solution (20 mL). Each mixture was subjected to 30 min ultrasonication to ensure homogeneous dispersion. The reduction process was initiated by adding 0.048 g of $NaBH_4$ to 10 mL $H_2O$ and taking 1 mL to the previous solution, then stirring was continued for 30 min to ensure complete reduction of the platinum precursor. The resulting products were collected by centrifugation, washed five times with deionized water to remove residual ions, and dried in a convection oven at 70 °C for 8 h, yielding the Pt@$MnO_2$ catalysts: Pt@Mn[α], Pt@Mn[β], Pt@Mn[γ], and Pt@Mn[δ].

*(3) Structural characterization*

The catalysts were characterized using a suite of analytical techniques. X-ray diffraction (XRD) patterns were acquired on a Bruker D8 Advance diffractometer equipped with Cu Ka irradiation (40 kV, 40 mA, $\lambda$=0.15418 nm), scanning over the 2θ range of 10 °to 80 ° at a rate of 5 °/min. Nitrogen adsorption–desorption experiments were measured at 77 K using the Quantachrome Autosorb-iQ-2 MP analyzer. Prior to analysis, samples were degassed at 300°C under dynamic vacuum for 6 h. Specific surface areas were calculated using the Brunauer–Emmett–Teller (BET) method, and pore size distributions were determined via the Barrett–Joyner–Halenda (BJH) model to the desorption branches of the isotherms. Fourier transform infrared (FT-IR) spectra were recorded on the Nicolet iS50 spectrometer over the range of 400 to 4000 $cm^{-1}$. Raman spectra were acquired using the LabRAM HR Evolution spectrometer with 532 nm laser excitation, scanning over the Raman shift range of 100 to 900 $cm^{-1}$. X-ray photoelectron spectroscopy (XPS) analysis was performed using the Thermo ESCALAB 250Xi. Binding energies were calibrated against the C 1s peak of adventitious carbon at 284.8 eV. UV-Vis DRS were conducted on the Shimadzu UV-2600 spectrophotometer equipped with an integrating sphere assembly. Optical bandgap energies were determined from Tauc plots using the following relationship: $\alpha$hv=Bd(hv-Eg), where $\alpha$, hv, Bd, and Eg represent absorption coefficient, photon energy, absorption constant, and bandgap energy, respectively. The exponent n depended on the nature of the semiconductor's optical transition: n=1/2 for direct bandgap semiconductors and n=2 for indirect bandgap semiconductors. Electrochemical impedance spectroscopy (EIS) and photocurrent response (IT) tests were performed using the CH1660C Apparatuses electrochemical workstation. EIS spectra were recorded at the open-circuit potential over a frequency range of $10^{-2}$ to $10^{-5}$ Hz. IT responses were measured under a bias potential of 0.8 V with simulated solar illumination. Hydrogen temperature-programmed reduction ($H_2$-TPR) and oxygen temperature-programmed desorption ($O_2$-TPD) experiments were performed on the ChemBET TPR/TPD analyzer. For $H_2$-TPR, samples were pretreated under $N_2$ flow at 150 °C for 1 h, cooled to 50 °C, then reduced in 5.0 vol% $H_2$/Ar flow while heating from 50 °C to 800 °C at a rate of 10 °C/min. For $O_2$-TPD, samples were pretreated in 30.0 vol% $O_2$/Ar at 300 °C for 1 h, cooled to room temperature, flushed with He for 30 min and heated from 30°C to 800°C at 10°C /min under He flow. Thermal desorption–gas chromatograph–mass spectrometer (TD-GC-MS) was employed to detect reaction byproducts in the reactor effluent. Exhaust gases were collected at different reaction temperatures using Tenax-TA tubes. These tubes were then thermally desorbed at 280 °C for 10 min

using the TurboMatri 350 (PerkinElmer, USA). The desorbed analytes were cryofocused in a trap and subsequently analyzed using an Agilent 7890A gas chromatograph coupled with a 5975C mass spectrometer detector (GC/MS, Agilent, USA).

*(4) Catalytic reaction*

The photothermal catalytic reactor was purchased from Hunan Huasi Instrument Co., Ltd. The catalytic performance was assessed in a quartz microreactor. The high-temperature reaction furnace measured 200 mm × 200 mm × 510 mm. The temperature range of the reaction furnace was from room temperature (RT) to 700°C, with a temperature control accuracy of < ±1°C. A K-type thermocouple with an outer diameter of 1.5 mm was used, featuring a special limit error of ±0.1°C. The light source (CEL-HXF300) was purchased from Beijing Perfectlight Technology Co.,Ltd, with the power density of of 297.3 mW·cm−2. Online monitoring of o-xylene and CO2 concentrations was conducted using a gas chromatograph equipped with a KB-5 capillary column and a flame ionization detector (FID). The reaction gas mixture, with a total flow rate of 50 mL/min, contained 300 ppm o-xylene in a synthetic air stream composed of 21 vol% O2 and 79 vol% Ar. A catalyst mass of 0.1 g, sieved to 20–40 mesh, was employed for the evaluation. The conversion of o-xylene (Xo-xylene) was calculated using the following equation.

$$X_{o\text{-}xylene}\,(\%) = (C_{in}\text{-}C_{out})/Cin\times100\%$$

Where X o-xylene was the o-xylene conversion; Cin and Cout referred to the concentration of o-xylene in inlet and outlet gas, respectively.

The catalyst stability test was assessed under the following conditions: in an atmosphere of 300 ppm o-xylene and air streams containing 79 vol% Ar and 21 vol% O2, at 110 °C. The test comprised alternating light/dark cycles: the catalyst was first illuminated for 10 h, followed by 10 h in the dark, and finally illuminated again for another 10 h. The o-xylene conversion was recorded throughout the entire duration of the stability test.

Reference:

1. Zhang, J.; Li, Y.; Wang, L.; Zhang, C.; He, H. Catalytic Oxidation of Formaldehyde over Manganese Oxides with Different Crystal Structures. Catal. Sci. Technol. **2015**, 5, 2305–2313, doi:10.1039/C4CY01461H.
2. Yang, W.; Su, Z.; Xu, Z.; Yang, W.; Peng, Y.; Li, J. Comparative Study of α-, β-, γ- and δ-MnO2 on Toluene Oxidation: Oxygen Vacancies and Reaction Intermediates. Appl. Catal. B Environ. **2020**, 260, 118150, doi:10.1016/j.apcatb.2019.118150.
